# Advancing liver cancer diagnosis and treatment with multi-omics approaches: a systematic review

**DOI:** 10.1007/s12672-025-03623-8

**Published:** 2025-11-24

**Authors:** Esraa M. Hashem, Ayat M. Karrar, Mai S. Mabrouk

**Affiliations:** 1 Biomedical Engineering Department, Misr University for Science and Technology (MUST University), 6th of October, Egypt; 2 System and Biomedical Engineering, City University of Cairo, New Heliopolis City, Badr, Egypt; 3 Center for Informatics Science (CIS), School of Information Technology and Computer Science, Nile University, 26th of July Corridor, Sheikh Zayed City, 12588 Giza Egypt

**Keywords:** Liver cancer, Multi-omics, Genomics, Proteomics, Transcriptomics, Metabolomics

## Abstract

Hepatocellular carcinoma (HCC), the most prevalent form of liver cancer, remains a major global health concern due to challenges in early detection and limited treatment options. Multi-omics technologies—such as genomics, proteomics, and metabolomics—enable comprehensive insights into the disease’s molecular complexity. This systematic review explores how these approaches contribute to biomarker discovery, molecular classification, and personalized treatment in HCC research. Methods: We conducted a structured review of 32 eligible studies, categorizing their computational methodologies into five primary analytical frameworks: survival analysis, unsupervised clustering, supervised machine learning, differential expression analysis, and pathway/network analysis. Notably, unsupervised clustering and supervised machine learning approaches, such as support vector machines, random forests, and deep learning models, were frequently used for subtype classification, feature selection, and predictive modeling. Results: The review identified that multi-omics approaches are widely used to discover biomarkers, classify HCC subtypes, and predict treatment responses. Common methods include clustering and machine learning. However, clinical validation remains limited, highlighting a gap in translational applicability. Conclusion: From a clinical perspective, multi-omics integration coupled with machine learning holds immense potential for improving early diagnosis, patient stratification, and therapeutic targeting. However, challenges related to data integration, interpretability, and cohort diversity must be addressed to realize this potential. This review underscores the transformative role of machine learning-enhanced multi-omics in reshaping liver cancer diagnosis and treatment and outlines future directions to bridge the gap between computational advances and clinical application.

## Introduction

The liver is a vital organ responsible for a variety of essential physiological functions, including metabolism, detoxification, and digestion. It processes nutrients, synthesizes proteins, and regulates biochemical homeostasis, making it indispensable to human health. Liver cancer is one of the most aggressive and lethal malignancies worldwide. It is the sixth most common cancer and the third leading cause of cancer-related deaths globally [1, 2]. The incidence of liver cancer varies significantly by region, with the highest rates in East and Southeast Asia, and parts of Sub-Saharan Africa, primarily due to the prevalence of hepatitis B virus (HBV) infection [3-5]. Other major risk factors include hepatitis C virus (HCV) infection, alcohol abuse, obesity, aflatoxin exposure, and non-alcoholic fatty liver disease. Liver cancer is more prevalent in males, with incidence rates nearly double those of females [6].

Liver cancer can be broadly classified into two categories: primary liver cancer, which originates in the liver, and secondary (metastatic) liver cancer, which spreads to the liver from other organs [7]. According to the 2019 World Health Organization (WHO) classification of liver and bile duct tumors, the main types of primary liver cancer (PLC) include hepatocellular carcinoma (HCC), intrahepatic cholangiocarcinoma (iCCA), combined hepatocellular–cholangiocarcinoma (cHCC-CCA), and hepatoblastoma [8]. This classification replaces earlier schemes that considered fibrolamellar hepatocellular carcinoma (F-HCC) a separate entity; it is now regarded as a histological variant of HCC due to overlapping molecular and pathological features.

## Subtypes of primary liver cancer

- Hepatocellular carcinoma (HCC): The most prevalent form of primary liver cancer, accounting for 75–85% of cases. It arises from hepatocytes and is commonly linked to chronic liver disease and cirrhosis. Only 5%–15% of patients are eligible for surgical resection due to late-stage diagnosis [9,6].
- Intrahepatic cholangiocarcinoma (iCCA): A malignancy originating from intrahepatic bile duct epithelium. It is the second most common type of PLC and is often diagnosed at an advanced stage due to its asymptomatic nature and molecular heterogeneity [2].
- Combined hepatocellular–cholangiocarcinoma (cHCC-CCA): A rare and aggressive tumor exhibiting both hepatocytic and cholangiocytic differentiation. It poses diagnostic and therapeutic challenges and has a poor prognosis [10].
- Hepatoblastoma: The most common liver cancer in children under five years of age. It is associated with developmental pathways, particularly dysregulation in Wnt/β-catenin signaling [11, 12].
- Fibrolamellar carcinoma (F-HCC): Now classified as a variant of HCC, not a distinct subtype. It typically affects adolescents and young adults without underlying liver disease and presents with distinct clinical and histological features [8].

One of the main challenges in treating liver cancer, especially HCC, is its significant intertumoral and intertumoral heterogeneity [13]. Genetic, epigenetic, and microenvironmental variability contributes to treatment resistance, disease progression, and relapse. Studies have shown that liver tumors often harbor multiple driver mutations and exhibit dynamic clonal evolution, making them particularly complex to diagnose and treat effectively [14, 15]. Conventional diagnostic methods, including imaging and serum biomarkers (e.g., AFP), often fail to detect early-stage liver cancer or differentiate between subtypes. This diagnostic gap results in late detection and limited treatment options, ultimately affecting patient survival. There is an urgent need for more precise molecular tools to improve early diagnosis, subtype classification, and therapeutic guidance.

In recent years, multi-omics technologies have emerged as essential tools for unraveling the biological complexity of liver cancer. These approaches allow for the comprehensive exploration of molecular alterations across various biological layers, offering deeper insights into tumor development, progression, and therapeutic vulnerabilities. Numerous molecular changes—spanning the genome, epigenome, transcriptome, proteome, metabolome, and microbiome—can act as indicators of malignant transformation. By leveraging affordable high-throughput technologies, researchers are now able to investigate these layers more extensively and in an integrated manner.

Among the omics domains, transcriptomics and proteomics have gained significant prominence due to their ability to capture dynamic regulatory and functional changes. Transcriptomics provides critical information at the regulatory level by analyzing RNA expression patterns. Through differentiation between protein-coding RNA (mRNA) and non-coding RNA (ncRNA), transcriptomics enables the identification of dysregulated pathways and genes associated with hepatocarcinogenesis [17]. Studying the transcriptome, the complete set of RNA transcripts produced under specific conditions—helps uncover disease mechanisms, discover biomarkers, and inform the development of targeted therapies in liver cancer.

**Fig. 1:**
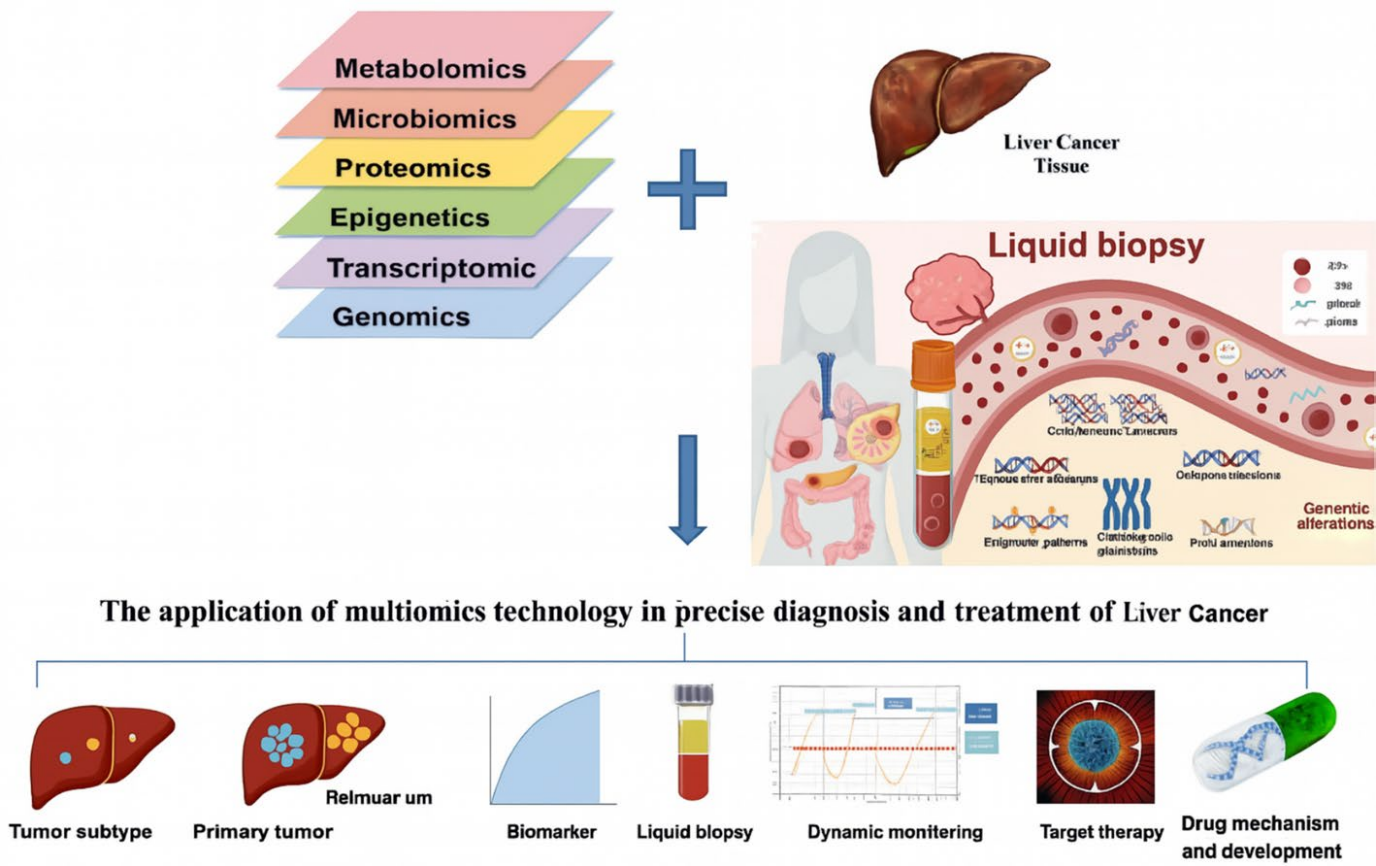
Multi-omics approaches in liver cancer

Complementing this, proteomics offers a functional perspective by examining the entire protein landscape within a biological system. It captures post-translational modifications, protein abundance, and interactions, providing insights beyond gene expression [18]. In liver cancer, proteomic approaches have been instrumental in identifying diagnostic and prognostic biomarkers, elucidating tumorigenic signaling pathways, and revealing potential therapeutic targets [19]. While alpha-fetoprotein (AFP) remains a standard biomarker, proteomics continues to drive the search for more accurate indicators. Additionally, proteomics facilitates the analysis of the tumor microenvironment—including secreted proteins and immune cell profiles—supporting the advancement of immunotherapies. Figure 1 illustrates the interconnected role of these omics’ technologies in liver cancer research [16].

Genomic studies, which examine an organism's complete DNA, have revolutionized our understanding of liver cancer. By identifying key driver genes, elucidating the mechanisms of tumorigenesis, and enabling personalized medicine approaches, genomics holds great promise for improving the diagnosis, treatment, and prognosis of liver cancer patients [20]. Large scale multi-omics analyses have provided a comprehensive landscape of genetic alterations in HCC [21].

Several genomic mutations drive the tumorigenesis and progression of HCC. Key driver mutations include those in the TP53 pathway, which governs genomic integrity and regulates cell growth; the WNT/β-catenin pathway, which modulates cell growth and tissue development; the NRF2/KEAP1 pathway, which modulates cellular response to oxidative stress; and the TERT promoter, which controls cell fate with the integration of genomic data with other omics technologies, HCC molecular alterations can be analyzed comprehensively, opening up new therapeutic approaches.

Biological metabolites are known as metabolomes, and the analysis of all these chemically heterogeneous small molecules is called metabolomics. Metabolomics can be performed with both targeted and untargeted approaches [22]. It provides a direct measure of the biochemical activity occurring within cells, reflecting the interplay between genes, proteins, and environmental factors. Using multi- omics scales to measure biological samples improves our understanding of the effects of genetic variations, the environment, and the interactions between them.

Handling the complexity of multi-omics data requires advanced computational techniques. Bioinformatics and machine learning (ML) approaches have become central to extracting meaningful insights from high-dimensional datasets. ML enables subtype classification, biomarker discovery, and predictive modeling for patient outcomes and drug response [20, 21]. However, their application in liver cancer research is still evolving and requires critical evaluation regarding reproducibility, interpretability, and clinical relevance.

Liver cancer is a severe and life-threatening disease that occurs when abnormal cells grow uncontrollably in the liver [23]. With the advancement of bioinformatics technology, the discovery and use of biomarkers for tumor detection, disease monitoring, and treatment have significantly increased. Bioinformatics technology can be leveraged to enhance the effectiveness of diagnosis and treatment for liver cancer by exploring molecular biomarkers closely associated with diagnosis, prognosis, and treatment response. In liver cancer research, multi-omics approaches have integrated proteomics, transcriptomics, metabolomics, genomics, and epigenomics. Since 2015, researchers have developed various methodologies to identify biomarkers, develop personalized and precision medicine, and understand disease mechanisms.

## Biomarker identification

When liver cancer reaches its middle or late stages, the best chances for treatment are often missed. Therefore, identifying biomarkers is essential for early detection, prognosis, and prevention. Biomarker discovery has become a central focus of research, especially with the widespread adoption of high-throughput multi-omics technologies and artificial intelligence (AI)-assisted data analysis. By integrating genomic, transcriptomic, proteomic, epigenomic, and metabolomic data, researchers can detect cancer related biomarkers with improved precision [24].

Specifically, PIWIL1 and PIWIL2, identified via transcriptomic profiling, are associated with HCC development and poor prognosis [34]. Aberrant DNA methylation patterns detected in ctDNA reflect epigenomic changes related to tumor progression [25]. Exosome proteins, derived from proteomic studies, offer diagnostic value, while multi-omics-based biomarkers, such as MCD-related genes, provide insights into mitochondrial dysfunction and cancer cell death [36].

Moreover, phosphoproteomics and AI-enhanced multi-omics analyses have revealed critical pathways and potential therapeutic targets, including MFAP5, PCNA, SOAT1, FGFR4, CDK1, and Src family kinases [29]. These discoveries highlight the growing role of multi-omics in driving biomarker discovery from exploratory research into actionable clinical applications [27].

Table 1 therefore supports and extends the narrative by offering concrete examples of approved and emerging biomarkers with established diagnostic or prognostic value in liver cancer.

Recent studies have demonstrated that integrative omics analyses can yield novel and clinically significant biomarkers for early-stage hepatocellular carcinoma (HCC)[28]. For example, circulating tumor DNA (ctDNA) mutations, changes in DNA methylation, and alterations in non-coding RNAs (e.g., lncRNAs, miRNAs) have been identified as promising diagnostic tools [30–33]. Likewise, exosome proteins identified through proteomics have been proposed as non-invasive biomarkers. These findings are summarized in Table 1, which categorizes representative biomarkers by their omics domain and outlines their relevance to HCC diagnosis and prognosis.

**Table 1:** Approved biomarkers for HCC

Biomarker	Omics category	Relevance to HCC	Reference
PIWIL2	Transcriptomics	Differential expressions associated with HCC development	[34]
PIWIL1	Transcriptomics	Overexpression linked to HCC progression and poor prognosis	[48]
cfDNA mutations	Genomics	Circulating tumor DNA mutations serve as non-invasive biomarkers for early HCC detection	[25]
Non-coding RNAs (e.g., lncRNAs)	Transcriptomics	Dysregulated lncRNA expression profiles associated with HCC; potential as blood-based biomarkers	[35]
Aberrant DNA methylation patterns	Epigenomics	Epigenetic alterations in circulating DNA are indicative of HCC presence	[26]
Exosomal proteins	Proteomics	Identified through multi-omics integration; potential for advanced diagnostic models	[32]
MCD-related genes	Multi-omics	Associated with mitochondrial cell death patterns; potential for prognostic modeling	[36]
PIWIL4	Transcriptomics	Altered expression patterns observed in HCC tissues	[34]

## Personalized and precision medicine

Despite major advancements in molecular oncology, many uncertainties remain about the underlying mechanisms of hepatocellular carcinoma. Multi-omics technologies have begun to fill these gaps by uncovering therapeutic targets and aiding in the development of personalized medicine frameworks. This integrative approach holds promise for improving therapeutic decision-making and tailoring treatment plans to individual tumor profiles [37].

The combination of genomic mutations, proteomic alterations, and transcriptomic deregulation enables clinicians to identify actionable molecular targets. For instance, mutations in MET and alterations in the FGFR signaling pathway have been found to predict response to targeted therapies. Such strategies can be especially effective in advanced HCC, where systemic therapy is standard but frequently limited by resistance.

Table 2 outlines a selection of approved targeted therapies and their associated molecular targets and pathways. For example, Lenvatinib targets multiple receptors including VEGFR1–3, FGFR1–4, and PDGFR, while Sorafenib inhibits key kinases such as RAF and VEGFR. These agents are linked to specific signaling pathways, such as Wnt/β-catenin, MAPK, and FGFR4, offering mechanistic precision in treatment approaches.

**Table 2:** Approved drugs and singling pathway of HCC

Target drugs	Target genes
Ramucirumab[42]	EGFR, ALK
Lenvatinib [43]	VEGFR-11, VEGFR-2, VEGFR-3, FGFR1, FGFR2, FGFR3, FGFR4, PDGFR, CKIT, RET
Sorafenib[44]	RAF kinase, EGFR-2, VEGFR-3, PDGFR-β, KIT, FLT-3/RAF/MEK/ERK
Donafenib [45]	VEGFR, PDGFR, RAF
Cabozantinib[46]	MET, VEGFR1/2/3, ROS1, RET, AXL, NTRK, KIT

Target drugs	Signaling pathway [47]
WNT/β-catenin	Celecoxib, PKF118-310, PKF115-584, CGP049090
RAF/MEK/ERK(MAPK)	Sorafenib, Regorafenib, Danoprevir/PD 0325901/PD 0325901
BLU-554	FGF19-FGFR4
Inhibitors of ARP	HRR

Beyond standard therapeutics, omics-based stratification models are also guiding next generation strategies. For instance, Zhiyong Wang et al. [39] developed a lysosomal gene signature (CTSV, LAPTM4B, DNAJC6, AP1M2) for clinical prediction. Xiaokai Yan [40] proposed m6A risk models for customizing chemotherapy and immunotherapy. Additional work by Yongfu Zhu [15] and Yitian Wei [41] provided insights into angiogenic signaling and oxidative stress responses using multi-omics profiling. These developments, combined with the drug target landscape in Table 2, illustrate how precision medicine in HCC is being shaped by integrative biological evidence.

## Disease mechanism discovery

Developing successful treatment plans requires an understanding of the pathogenic mechanisms underlying the development of HCC with cirrhosis [6]. Multi-omics approaches have significantly advanced our understanding of the complex biological processes underlying liver cancer. Researchers can clarify the disease pathways that contribute to tumor initiation, development, and therapeutic resistance by combining data from many omics’ levels.

### Tumor heterogeneity and evolution

Multi-omics data reveal the genetic and epigenetic diversity within liver tumors. This heterogeneity contributes to the differential responses to therapy and disease progression [47]. Single-cell multi-omics studies have provided insights into clonal evolution and the emergence of therapy-resistant subpopulations [49]. By understanding tumor heterogeneity, personalized treatment plans can be developed to target distinct subclones, thereby reducing the likelihood of resistance and improving patient outcomes [50].

### Signaling pathway dysregulation

Integrative analyses of genomics, transcriptomics, and proteomics data have uncovered the key signaling pathways driving liver cancer. Furthermore, while studies often focus on the molecular hallmarks of HCC, they rarely contextualize findings within broader oncological trends. For example, the deregulation of pathways such as PI3K/AKT/mTOR and Wnt/β-catenin is not unique to liver cancer [51]. In addition, aberrant Notch and Hippo signaling pathways have been implicated in promoting HCC initiation and progression, presenting further avenues for therapeutic intervention [52]. Comparative pan-cancer analyses using multi-omics could illuminate both shared and liver specific vulnerabilities, yet such syntheses are currently scarce. This presents a key opportunity for future research to position liver cancer within the broader landscape of molecular oncology, potentially revealing universal therapeutic targets or resistance mechanisms.

### Metabolic reprogramming

Metabolic reprogramming is a hallmark of liver cancer. Tumors exhibit increased lipid biosynthesis and alter glucose metabolism. Understanding these metabolic changes provides opportunities to target tumor specific metabolic vulnerabilities [50]. For example, inhibiting glutamine metabolism or modulating the Warburg effect has emerged as a promising approach for treating metabolically driven HCC [53].

### Immune microenvironment modulation

Multi-omics approaches have revealed how liver cancer modulates the immune microenvironment to evade immune surveillance [51]. Transcriptomic and proteomic analyses of tumor-infiltrating immune cells have identified potential biomarkers for the immunotherapy response and resistance mechanisms [53]. Furthermore, single-cell RNA sequencing has elucidated the role of exhausted T cells and immunosuppressive macrophages in limiting the efficacy of immune checkpoint inhibitors [53].

### Epigenetic regulation

Epigenomic profiling has identified aberrant DNA methylation and histone modifications that contribute to liver cancer progression [53]. These epigenetic changes regulate key oncogenes and tumor suppressor genes, presenting novel therapeutic targets for epigenetic drugs [54]. For example, inhibitors targeting DNA methyltransferases (DNMTs) and histone deacetylase (HDACs) are under investigation to reverse epigenetic dysregulation and restore normal gene expression [50].

Because HCC is such a complex and heterogeneous disease, ideal analytical platforms are urgently needed to identify the molecular mechanisms underlying liver cancer to facilitate more effective diagnostics and therapeutics. Over recent years, multi-omics approaches, combining genomics, transcriptomics, proteomics, and metabolomics, have become a panacea to help decode the biological complexities that underpin HCC and other subtypes of liver cancer. Such methods can generate more information on tumor heterogeneity, disease progression and treatment response. Nevertheless, dealing with the large amounts and dimensionality of omics data requires the development of sophisticated computational methods for robust integration and interpretation. In this sense, this work aims to provide a systematic overview of their current applications, with an emphasis on the ways in which statistical and machine learning methodologies have been utilized for biomarker discovery, patient stratification, and therapeutic target identification in liver cancer research. Integration of these observations, which encompass network-based findings, represents the primary objective of this review, to delineate current methodological trends, evaluate existing shortcomings, and stimulate approaches to translational and precision hepatology.

## Methods

To ensure transparency, a comprehensive analysis of existing research was conducted following the well-established Preferred Reporting Items for Systematic Reviews and Meta-Analyses (PRISMA) [55].

### Search strategy and inclusion criteria

The systematic review was conducted according to the Preferred Reporting Items for Systematic Reviews and Meta-Analyses (PRISMA) guidelines [55]. To assess the latest research in the context of multi-omics in liver cancer, a comprehensive search for relevant studies was conducted. The search was conducted electronically to select papers published in the previous 10 years in the PubMed and google scholar database.

The keywords and search algorithms employed to refine the selection of articles that are significant to this study are presented below. (“Liver cancer” OR “Neoplasm, Hepatic” OR “Hepatic Cancers” OR “Hepatocellular Cancers” OR “Hepatocellular Carcinoma” OR “Liver Cell Carcinomas”) AND (“Sc-RNAseq” OR “Single-Cell RNA-Seq” OR “Single Cell RNA Seq”) AND (“Multi-Omics” OR “Integrative-Omics”).

**Fig. 2:**
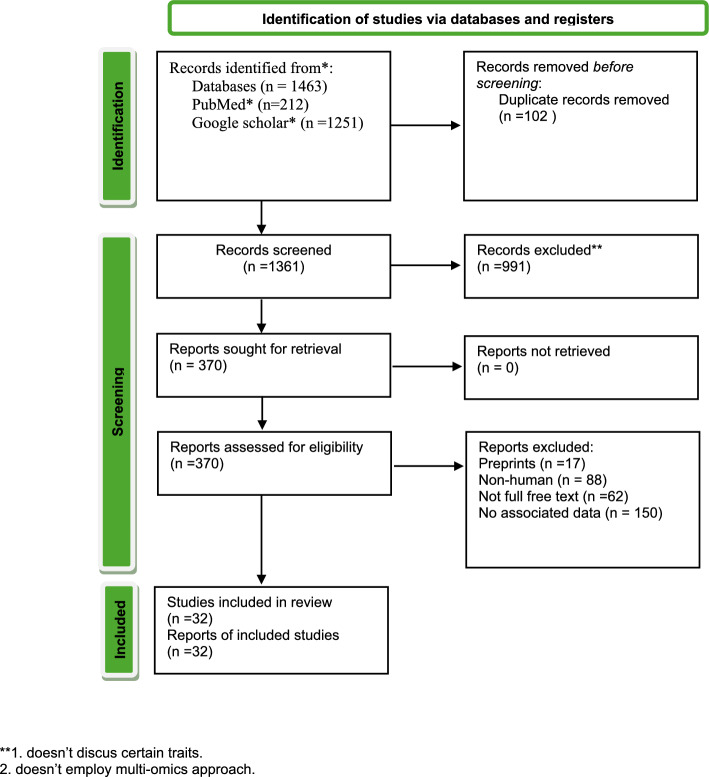
PRISMA 2020 flow diagram for new systematic reviews. **1. Doesn’t discus certain traits. 2. Doesn’t employ multi-omics approach

The inclusion criteria were: (1) studies involving human subjects, (2) application of multi-omics analysis, and (3) original research articles. Exclusion criteria included reviews, editorials, animal only studies, and studies lacking integrative omics approaches. Figure 2 presents the PRISMA flowchart summarizing the article selection process.

The methodological quality of the included computational and multi-omics studies was assessed using an adapted version of the *Quality In Prognosis Studies (QUIPS)* tool. This framework was modified to address aspects specific to in-silico research. Five domains were evaluated for each study: (i) data source and cohort quality (sample size, representativeness, and data completeness); (ii) reproducibility and transparency (availability of data, code, and clear workflow descriptions); (iii) validation and robustness (use of internal cross-validation, external datasets, or experimental confirmation); (iv) appropriateness of computational and statistical methods (suitability of algorithms and integration techniques for multi-omics data); and (v) reporting quality (clarity of presentation, linkage between methods and conclusions, and acknowledgement of limitations). Each study was reviewed independently across these domains, and qualitative ratings were summarized in Table 3.

**Table 3:** QUIPS-based quality assessment of included studies

Study (Ref)	Analysis type/approaches	Multi-omics technology	Data source & cohort quality	Reproducibility/transparency	Validation/robustness	Appropriateness of methods	Reporting quality
Hongyu Chen [56]	Radiomics, factor analysis, ML	scRNA-seq, TCM dataset	Moderate—1445 prescriptions; culturally specific dataset	Limited—Proprietary software, unclear reproducibility	Limited—No external validation	Appropriate—Frequency & factor analysis match goals	Clear but context-specific
David Requena [57]	Epigenetics (PKA signaling)	RNA-seq, methylation	High—1412 RNA-seq samples	Good—Workflow documented with Nextflow	Moderate—Mostly computational validation	Appropriate bioinformatics pipeline	Well-documented
Liu Y. [60]	Pathway & survival analysis	CNV, transcriptome, proteome	Moderate—80 patients	Limited—Code not publicly available	Limited—Validation on single dataset	Suitable—Standard survival models	Clear
Zhiyong Wang [39]	ML immune deconvolution	scRNA-seq	High—GDC data	Good—Tools (CIBERSORT, EPIC) well documented	Good—Multiple algorithmic validations	Advanced ML, appropriate	Well-presented
Xu L. [24]	Multi-omics survival analysis	scRNA-seq, transcriptomic, methylation	High—TCGA + GEO datasets	Good—Public datasets	Good—Multivariate Cox regression validated	Comprehensive	Clear with good clinical link
Lu CY [64]	ML with graph neural network	DNA methylation	High—TCGA	Good—Reproducible ML methods	Moderate—Mostly computational validation	Advanced GNN appropriate	Clear
Yanli Zhang [65]	Splicing network & prognosis	RNA-seq	High—TCGA + GEO	Good—Publicly available	Moderate—Model validated on subsets	Standard regression & pathway methods	Clear
Ye J [66]	Differential expression, clustering	scRNA-seq, ST-seq	Very high—7269 HCC patients	Good—Methods documented	Strong—Multi-dataset validation	Robust	Excellent
Yuqi Miao [68]	ML clustering	Multi-RNA types	High—TCGA	Good—ML workflow described	Moderate—Subtype validation in silico	Advanced ML	Clear
Li S [69]	Immune pathway analysis	scRNA-seq, proteomics	High—TCGA + GEO	Good	Good—Drug response validated computationally	Suitable	Clear
Li J [70]	Gene expression & immune infiltration	mRNA	High—TCGA + GEO	Good	Moderate	Suitable	Clear
Li Chen [71]	Multi-omics ML	transcriptome, proteomics	High	Good—NMF, RF methods documented	Good—Cross-validation	Appropriate ML	Clear
Yong Pan [72]	Drug sensitivity, survival	scRNA-seq	High	Good	Good	Suitable	Clear
Xiaokai Yan [37]	m6A risk modeling	scRNA-seq	High	Good	Moderate	Suitable	Clear
Yang Li [73]	Iron metabolism analysis	RNA-seq, scRNA-seq	High	Good	Good	Suitable	Clear
Xuan M [41]	WGCNA, enrichment	RNA-seq	High	Good	Moderate	Appropriate	Clear
Ping Sun [76]	117 ML models	scRNA-seq	High	Good	Strong—Multiple models tested	Advanced ML	Clear
Y. Zhu [39]	ML, SCENIC	scRNA-seq	High	Good	Strong—Cellchat & SCENIC validated	Advanced	Clear
Fan M [78]	LC–MS + multi-omics	Proteomics, metabolomics	Moderate—14 pairs	Limited	Limited—No external validation	Suitable	Clear
Xiangzhan Kong [79]	Pathway, ML	scRNA-seq, proteomics	High	Good	Good	Suitable	Clear
Xiu-Ping Zhang [81]	Metabolic, epigenetic	scRNA-seq	High	Good	Moderate	Suitable	Clear
Junhong Chen [83]	GSVA, ML	scRNA-seq	High	Good	Good	Suitable	Clear
Chunqing Wang [84]	Epigenomics	scRNA-seq, chromatin	High	Good	Limited	Advanced	Clear
Yan-zhu Chen [87]	Multi-omics integration	Bulk RNA-seq, scRNA-seq	High	Good	Good	Suitable	Clear
Yitian Wei [40]	Graph neural networks	RNA-seq, scRNA-seq	High	Good	Strong—External validation	Advanced	Clear
Xiaoyan Wang [88]	Kinase pathway	RNA-seq	High	Good	Good	Suitable	Clear
Leonie Wuerger [89]	MR, GWAS	scRNA-seq, RNA-seq	High	Good	Good	Appropriate	Clear
Yanteng Zhao [91]	Differential methylation	RNA-seq	Low—12 patients	Limited	Limited	Suitable	Clear
Yan He [38]	Prognostic models	scRNA-seq	High	Good	Moderate	Appropriate	Clear
Chengbang Wang [32]	ML diagnostic model	scRNA-seq	High	Good	Good	Appropriate	Clear
Rui Fan [92]	Immune analysis	scRNA-seq	High	Good	Moderate	Suitable	Clear
Zhen Liu [93]	ECM analysis, ML	scRNA-seq	High	Good	Good	Advanced ML	Clear

Overall, the quality assessment indicated that the majority of studies demonstrated a low to moderate risk of bias, with strong methodological rigor in terms of data processing and analytic approaches. Weaknesses were mainly observed in external validation, lack of publicly available code, and limited reproducibility in studies relying on proprietary software or small sample sizes. These limitations highlight the need for standardized protocols and reproducible workflows in computational multi-omics research.

### Data collection

The relevant data were extracted from the articles after performing a qualitative screening of publications and acquisition of related research that satisfied the inclusion requirements. The subsequent information was collected from every article: year of publication, investigated trait, type of data used, multi-omics approach, and their findings.

## Results and discussion

Three hundred and seventy references were retrieved from the PubMed and Google Scholar databases. After performing a preliminary assessment of each publication, 317 articles were eliminated because they failed to fulfill the inclusion requirements. After evaluating 53 suitable full-text references, 21 proved irrelevant and were eliminated. Eventually, 32 articles were chosen for final evaluation based on the previously demonstrated eligibility criteria. Figure 2 illustrates the criteria used for research inclusion. Researchers conducted different multi-omics approaches to identify biomarkers, develop personalized treatments, understand disease mechanisms, and predict the treatment outcomes of liver cancer. Table 4 presents the findings and methods employed in the chosen studies.

**Table 4:** Findings, bioinformatic analysis and methods employed in the chosen studies

Study	Analysis type	Tool	Dataset	Multi-omics technology	Approaches	Findings and insights
Hongyu Chen. [56]	Radiomics, frequency analysis, factor analysis, and ML	IBMSPSSModeler18software	1,445 TCM prescriptions and 188 different herbs used in liver disease treatment. [56]	scRNA-seq	Personalized and Precision Medicine	Tonic deficiency medicine, solution table medicine, water infiltration wet medicine, qi medicine, blood circulation and blood stasis medicine, li medicine, and liver xifeng medicine are the most prevalent drugs
David Requena. [57]	PKA signaling pathway	Nextflow, yaml, cutadapt, Trim Galore, and Bismarck	1412 RNA-seq samples. [58, 59]	RNA-seq, transcriptomic	Disease Mechanism Discovery	22 Protocadherin family cluster genes (PCDHGA1 to 8, PCDHGB1 to 5, and PCDHA1 to 9) were found to be hypermethylated, and FOXA1 and FOXA2 were shown to be downregulated in FLC
Liu Y. et al. [60]	Pathway analysis	Kaplan–Meier curves and Cox proportional hazards models, and CCK8	80 patients with liver cancer [60]	Genomic CNV, transcriptome and proteome analysis	Disease Mechanism Discovery	BAZ2A transcription levels were highest in LM6 cells, and BAZ2A expression was higher in LIHC tissues than in normal tissues
Zhiyong Wang. [39]	Machine learning	CIBERSORT- ABS, MCpCOUNTER, QUANTISEQ, XCELL, EpIC algorithms	GDC database [61]	scRNA-seq	Biomarker Identification	shown that in vitro, CTSV gene knockdown significantly decreased HCC migration and proliferation
Xu L [24]	Kaplan–Meier Survival Analysis, Cox Proportional Hazards Regression, and KEGG Pathway Analysis	ESTIMATE algorithm	The Cancer Genome Atlas (TCGA) [62] and the Gene Expression Omnibus (GEO)[63]	scRNA-seq, transcriptomic, genomic, and methylation data	Personalized and Precision Medicine	According to these findings, KLHL23 may be a biomarker for a number of cancer forms, particularly LIHC
Lu CY.[64]	Computational analysis, ML	Multi-level attention graph neural network (MLA-GNN)	TCGA [62]	DNA methylation	Biomarker Identification	Lenvatinib is an established and promising drug for the treatment of advanced hepatocellular carcinoma
Yanli Zhang [65]	KEGG pathway enrichment analyses	Cox proportional hazards regression and developed a splicing network	TCGA [62] and GEO [63]	RNA-seq	Personalized and Precision Medicine	Highlights FTCD’s potential impact on HCC clinical diagnosis and treatment strategies
Ye, J [66]	Differential gene expression analysis, Pathway analysis, and Clustering analysis	CASAVA, CopyKAT	7269 HCC patients from the databases of Cancer Genome Atlas Liver Hepatocellular Carcinoma (TCGA-LIHC) [67]	scRNA-seq, ST-seq	Disease Mechanism Discovery	The data reveals how important HCC cells are in creating dynamic tumor ecosystems that match clinical situations
Yuqi Miao, [68]	Machine learning	PartIES,	TCGA [62]	mRNAs, lncRNAs, and miRNAs	Disease Mechanism Discovery	Identified cancer subtypes of HCC have different activity levels for some known cancer-related pathways
Li S [69]	Immune signaling pathways	CellChat, CIBERSORT	TCGA [62] and GEO [63]	scRNA-seq, ST-seq, proteomic analysis	Personalized and Precision Medicine	Drug sensitivity analyses indicated that TP-TME high-risk subtypes, including sorafenib and pembrolizumab, were associated with sensitivity to multiple drugs
Li J [70]	Gene Expression Analysis, Immune Cell Infiltration Analysis, and Gene Ontology (GO) and KEGG Pathway Analysis	GEPIA, CIBERSORT	TCGA [62] and GEO [63]	mRNAs	Disease Mechanism Discovery	CDK1 and DLGAP5 were shown to be associated with tumor immune cell infiltration promoting HCC progression
Li Chen [71]	Integrated Multiomics Analysis, Programmed Cell Death (PCD) Analysis, and Gene ontology (GO) and KEGG pathway enrichment analyses	Elastic net, Random Forest, XgBoost, and Boruta, Nonnegative Matrix Factorization, NMF	TCGA [62] and GEO [63]	transcriptomic, proteomic and scRNA-seq	Disease Mechanism Discovery	The results acknowledged the wide range of molecular subtypes linked to PCD
Yong Pan [72]	Kaplan–Meier survival analysis, Immunotherapy Efficacy Analysis, Drug Sensitivity Analysis	TIMER, XCell, MCPCOUNTER, CIBERSORT, EPIC	TCGA [62] and GEO [63]	scRNA-seq	Biomarker Identification	The results of the molecular docking research showed that 5-fluorouracil, gemcitabine, paclitaxel, and sorafenib had excellent binding affinities and that PDHA1 expression was strongly correlated with their sensitivity
Xiaokai Yan [37]	N6-methyladenosine (m6A) Regulator Analysis, and Tumor immune microenvironment analysis	CIBERSORT, GSVA’s	TCGA [62] and GEO [63]	scRNA-seq	Biomarker Identification	The discovery of m6A risk models may help inform decisions about immunotherapy, targeted therapy, and customized chemotherapy for HCC
Yang Li [73]	Integrated Multi-omics Analysis, Iron Metabolism Pathway Analysis and Immune Microenvironment Analysis	Iron metabolism genomic scoring, CIBERSORT	TCGA [62] and GEO [63] RNA-seq data for HCC was downloaded from the UCSC Xena platform [74]	scRNA-seq	Disease Mechanism Discovery	Iron metabolism abnormalities are not only drivers of liver cancer development but also key indicators of patient prognosis
Xuan, M [41]	Single-Sample Gene Set Enrichment Analysis (ssGSEA), Weighted Gene Co-Expression Network Analysis, and Cell–Cell Communication Analysis	ssGSEA, MEbrown and MEgreen	UCSC Xena platform [73], 50 hallmark genes and their related gene symbols were obtained from the Molecular Signatures Database [75]	RNA-seq	Disease Mechanism Discovery	The study revealed two gene module eigengenes (MEs) associated with cirrhosis, namely, MEbrown and MEgreen
Ping Sun [76]	117 machine learning methods,	HMRS	445 HCC samples with prognostic data from the International Cancer Genome Consortium (ICGC) [77] and [62, 63]	scRNA-seq	Personalized and Precision Medicine	The HMRS offers insights into underlying molecular mechanisms, immune characteristics, and potential therapeutic strategies
Y. Zhu [39]	Machine Learning Methods, Cellchat analysis, SCENIC analysis	pySCENIC, Scrublet, Stepwise Cox, CoxBoost, Cox Partial Least Squares Regression (plsRcox), Supervised Principal Component (SuperPC)	GEO [63]	scRNA-seq, transcriptomic	Personalized and Precision Medicine	Apelin may serve as a potential therapeutic target in HCC
Fan M [78]	Ingenuity Pathway Analysis (IPA)	Liquid chromatography-mass spectrometry (LC–MS)	14 pairs of HCC and corresponding non-tumor liver tissue samples [78]	proteomics, phosphoproteomics, metabolomics, and lipidomics	Disease Mechanism Discovery	The proposed specific potential biomarker panels(ROCK1 deactivation and GSK3A activation for both early and advanced-stage HCC
Xiangzhan Kong [79]	single-sample gene set enrichment analysis (ssGSEA), Differential gene expression analysis, Pathway analysis, and ML models	GSVA algorithm, *t*-test, Pearson correlation coefficient, Log-rank (Mantel–Cox) test, and One-way ANOVA	TCGA [62], GEO [63] and 1374 patients diagnosed with HCC [80]	scRNA-seq, transcriptomic, proteomics	Disease Mechanism Discovery	The identification of TPC-related gene profiles helps forecast treatment results and patient outcomes
Xiu-Ping Zhang [81]	Metabolic pathway analysis, Meta-cluster calculation, Differential gene expression analysis,	REACTOME and GSVA, and pySCENIC method	GEO [63], and ICGC database (ICGC‐LIRI‐JP) [82]	scRNA-seq,	Disease Mechanism Discovery	Discover new directions for understanding disease development and immunotherapy responses
Junhong Chen [83]	Gene set variation analysis (GSVA), Differential gene expression analysis, Pathway enrichment analysis, and ML	TIMER, QUANTISEQ, MCPCOUNTER, XCELL, EPIC, and CIBERSORT	TCGA [62] and GEO [63]	scRNA-seq, transcriptomic,	Disease Mechanism Discovery	The discovery of the ADAM gene family's molecular heterogeneity, underscoring its important role in the onset and spread of HCC
Chunqing Wang[84]	Analysis of chromatin accessibility and histone modification patterns, Tn5 transposases	multi-modal scCPA-Tag, ChIPseeker	CNGB Sequence Archive (CNSA) [85] of China National GeneBank DataBase (CNGBdb) [86]	scRNA-seq,	Disease Mechanism Discovery	The suggested model offers a thorough method for investigating the diverse cell types' epigenetic landscapes
Yan-zhu Chen[87]	Tumor microenvironment analysis, Differential gene expression analysis, and Pathway enrichment analysis	scRNAtool, VECTOR, and CellChat	TCGA [62], GEO [63], TCGA-LIHC [67]	Bulk RNA-seq, scRNA-seq, stRNA-seq	Disease Mechanism Discovery	According to the suggested paradigm, HMGB2 plays a crucial role in the development, metastasis, and immunosuppression of HCC
YitianWei[40]	Multi-Layer Attentive Graph Neural Network, ML, Differential expression and methylation analysis	MLA-GNN, XGBoost, DT, GBM, SVM	GEO [63], TCGA-LIHC [67]	RNA-seq, scRNAseq, stRNA-seq	Disease Mechanism Discovery	For HCC patients, the OSMTS results show promise as a tool for accurate intervention and prognosis prediction
Xiaoyan Wang [88]	Pathway analysis, Kinase-substrate enrichment analysis	Genome- metabolome-Mendelian Randomization, DigiWest	GEO [63]	RNA-seq	Disease Mechanism Discovery	The findings showed the value of an integrated genome-metabolome-Mendelian Randomization strategy for predicting disease progression across several databases
Leonie T. D. Wuerger [89]	Mendelian Randomization, Genome-wide association studies (GWAS) analysis	clusterProfiler, MR-Egger	GWAS Catalog [90], GEO [63],	scRNAseq, RNA-seq	Biomarker Identification	The outcomes demonstrate the role of genes linked to retinol metabolism (ADH1A, CYP2A6, CYP2C8, and CYP2C19) in the onset and spread of HCC
Yanteng Zhao [91]	Differential methylation analysis, Correlation analysis between methylation and gene expression	RSEM, ESTIMATE tool	12 HCC patient [91], TCGA [62], GEO [63]	RNA sequencing, shotgun proteomics, transcriptomics	Disease Mechanism Discovery	The findings improve our understanding of the molecular importance of OA and its possible effects on health
Yan He [38]	Gene set enrichment analysis, Pathway enrichment analysis	multivariate Cox regression and LASSO regression	TCGA [62], GEO [63]	scRNAseq	Personalized and Precision Medicine	The findings demonstrated RPN1's molecular roles in liver cancer and raised the possibility that it could be used as a therapeutic target for the condition
Chengbang Wang. [32]	Differential gene expression analysis, ML algorithms for model development, and Pathway enrichment analysis	exoRBase HCC, GEPIA2.0	GEO [63], TCGA-LIHC [67]	scRNA-seq, RNA-seq, stRNA-seq	Personalized and Precision Medicine	With TRIB3 emerging as a promising diagnostic tool and therapeutic target for HCC
Rui Fan. [92]	Gene Set Enrichment Analysis (GSEA), Immune cell infiltration analysis. and Differential gene expression analysis	lentiCRISPR-EGFP sgRNA	GEO [63], TCGA-LIHC [67]	scRNA-seq	Personalized and Precision Medicine	Important new information on KHDRBS1 as a therapeutic target in HCC is provided by this study
Zhen Liu. [93]	Differential gene expression analysis, pathway enrichment analysis, ML algorithms for model development, and extracellular Matrix related genes and pathways	Ggpubr, xCELL,	GEO [63], TCGA-LIHC [67]	scRNA- seq	Disease Mechanism Discovery	Suggested that ECM may have an impact on tumors that goes beyond merely encouraging the fibrotic process and the stromal makeup of tumors

Hongyu Chen et al. [56] used multi-omics to Personalized Treatment of liver cancer. They are studying the composition of Traditional Chinese Medicine (TCM) compounds and their mechanism of action at the molecular level. Combining imaging omics with multi-omics characteristics and integrating with the omics of TCM prescriptions can more comprehensively reveal the biological characteristics of tumors. The study results showed that a total of 1445 prescriptions were selected to treat liver disease, involving 188 kinds of Chinese medicine, among which the most frequently used single medicine was Angelica (688 times), followed by Licorice (687 times). They successfully concluded the drugs are the most abundant, followed by tonic deficiency medicine, solution table medicine, water infiltration wet medicine, qi medicine, blood circulation and blood stasis medicine, li medicine and liver xifeng medicine, etc.

Requena et al. [57] conducted a comprehensive multi-omics analysis of 1,412 liver cancer samples to understand the molecular features of FLC and its relation to other liver cancers. Their study identified a 693-gene FLC-specific signature, distinguishing it from typical HCC. Similarly, Liu et al. [60] analyzed tumor tissues from 80 liver cancer patients and found that the expression of BAZ2A was significantly higher in tumors than in surrounding tissues. This overexpression was associated with better prognosis in several types of cancer, including HCC.

Wang et al. [39] developed a lysosome-associated prognostic treatment index (LAPTI) using machine learning, based on four lysosome-related genes. They found that a high LAPTI score was associated with poor prognosis and increased immune cell infiltration in HCC. Xu et al. [24] identified KLHL23 as a potential biomarker for cancer prognosis, showing that lower expression of this gene was linked to higher immune activity and increased chemotherapy sensitivity. Lu et al. [64] highlighted the diagnostic and therapeutic potential of FOXL2 through gene ontology and molecular docking analyses, particularly in relation to the liver cancer drug lenvatinib.

Zhang et al. [65] explored the role of the FTCD gene and alternative splicing events in a cohort of 343 HCC patients. Their findings suggested that RNA splicing patterns are associated with patient prognosis and can guide future treatment strategies. Ye et al. [66] mapped the spatiotemporal landscape of HCC heterogeneity, identifying niche genes related to tumor recurrence and confirming their relevance in a large, multi-cohort validation of over 7,000 patients.

Miao et al. [68] proposed a novel multi-omics integration framework that preserved omics-specific clustering structures and identified subtypes enriched with essential cancer genes and pathways. Li et al. [69] further categorized HCC subtypes with distinct molecular features and drug sensitivities, noting that high-risk groups had elevated immune checkpoint markers and were more responsive to therapies like sorafenib and pembrolizumab. Likewise, Li et al. [70] associated CDK1 and DLGAP5 with immune infiltration and tumor progression in HCC.

In an effort to personalize treatment, Chen et al. [71] developed a programmed cell death index (PCDI) using five robust gene signatures across four independent HCC cohorts. Pan et al. [72] demonstrated that high PDHA1 expression predicted poor survival and served as an independent prognostic factor for HCC. Yan et al. [37] focused on m6A RNA methylation regulators and constructed risk models that could aid in selecting immunotherapy and chemotherapy regimens for HCC patients.

Li et al. [73] studied the impact of iron metabolism on patient survival and showed that iron metabolism-related genes could serve as prognostic indicators.

Xuan et al. [41] used gene co-expression analysis to identify two modules—MEbrown and MEgreen—linked to cirrhosis in liver cancer. Sun et al. [76] developed a histone modification-related prognostic model (HMRS) using machine learning, integrating multi-omics and clinical data to stratify patients based on survival and drug response.

Zhu et al. [39] evaluated the role of the apelin/APJ pathway in angiogenesis and demonstrated that silencing apelin reduced tumor blood vessel formation, suggesting its value as a therapeutic target. Fan et al. [78] performed multi-omics profiling of early and advanced-stage HCC, identifying biomarkers and pathways like ROCK1 and GSK3A involved in tumor progression. Kong et al. [79] constructed a risk model based on TPC-related genes involved in immune pathways and vascular remodeling, which was predictive of treatment outcomes.

Zhang XP et al. [81] studied metabolic heterogeneity between HCC and portal vein tumor thrombus (PVTT), finding that polyamine metabolism was a key altered pathway. Chen J et al. [83] used a machine learning-based multi-omics approach to highlight the prognostic value of ADAM family genes, achieving high prediction accuracy. Wang C et al. [84] developed a novel single-cell profiling method (scCPA-Tag) to assess chromatin accessibility and histone modifications simultaneously, revealing key epigenetic mechanisms in HCC cells. Chen YZ et al. [87] integrated bulk and single-cell RNA sequencing with spatial transcriptomics and clinical validation to identify HMGB2 as a key factor in HCC progression and immune suppression. Wei Y et al. [40] constructed a 26-gene oxidative stress mitochondria-related signature (OSMTS) predictive of prognosis and treatment response in HCC. Wang X et al. [88] used Mendelian randomization and network analysis across large public datasets to create a 12-gene fibrosis prediction model applicable across populations.

Wuerger et al. [89] examined the effects of Okadaic acid (OA) using transcriptomic, proteomic, and phosphoproteomic data, uncovering systemic impacts on liver cell metabolism, cytoskeletal structure, and signaling via PP2A suppression. Zhao Y et al. [91] profiled DNA methylation and gene expression in HCC tissues and identified retinol metabolism-related genes as potential diagnostic and therapeutic targets. He Y et al. [38] analyzed over 10,000 samples to characterize disulfidptosis-related genes (DRGs) in pan-cancer, developing a predictive model and identifying RPN1 as a therapeutic target in liver cancer.

Finally, Wang C et al. [32] built a seven-gene diagnostic model for HCC using single-cell and bulk transcriptomic data, identifying TRIB3 as a potential therapeutic target. Fan R et al. [92] showed that KHDRBS1 promotes tumor growth in HCC, supported by molecular biology and single-cell sequencing. Liu Z et al. [93] used single-cell transcriptomics and functional assays to show that extracellular matrix-related genes influence immune cell distribution and tumor behavior in HCC, providing new insight into the tumor microenvironment.

**Fig. 3:**
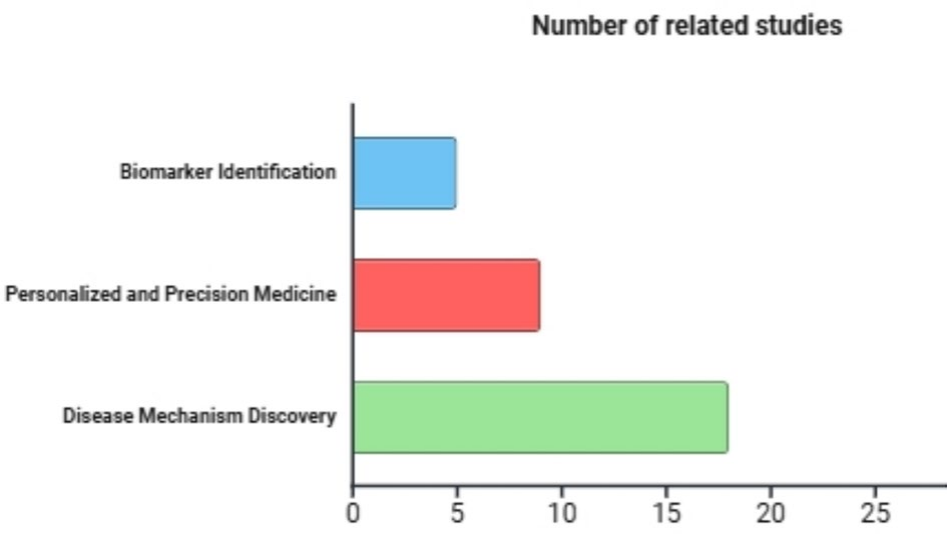
Number of the multi-omics approaches employed in the chosen research

**Fig. 4:**
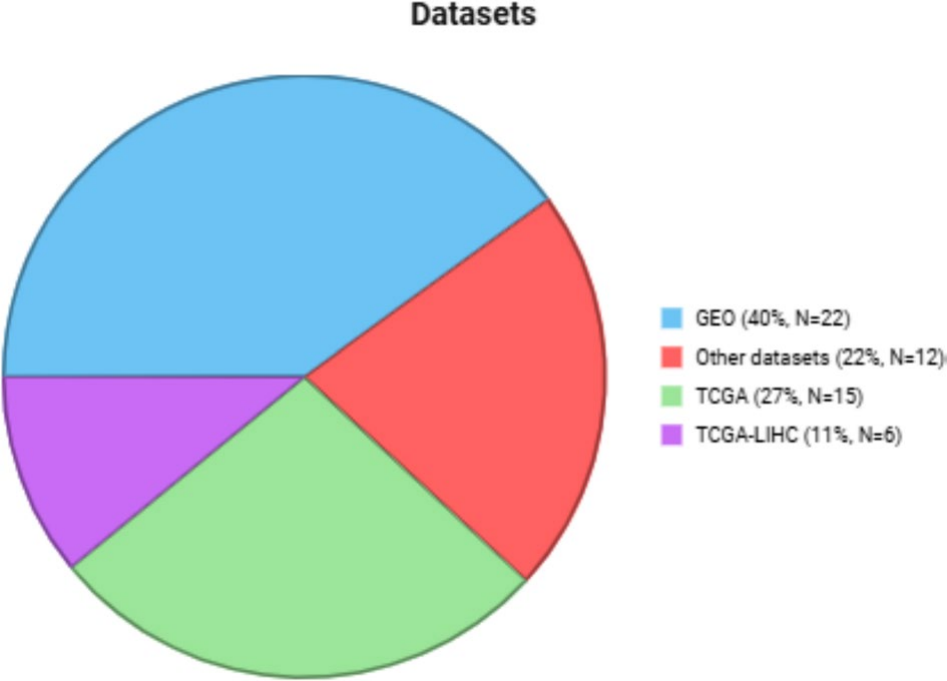
Summary of the datasets used in the reviewed studies

The distribution of 32 studies across the areas of biomarker identification, personalized medicine, and disease mechanism discovery is visualized in Fig. 3. Figure 4 displays the percentage of studies that utilize the most frequently selected datasets. Gene Expression Omnibus is the most dataset used in 40% of the 32 studies.

**Fig. 5:**
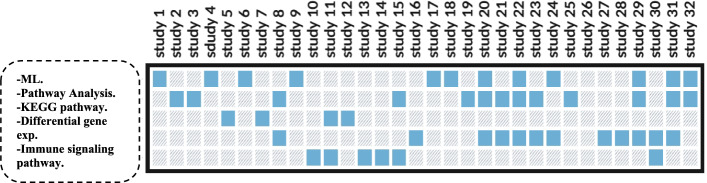
Summarize the selected studies by categorizing them based on the types of analyses conducted

Figure 5, it is evident that the most commonly utilized bioinformatics multi-omics analyses in liver cancer include machine learning (ML), pathway analysis, differential gene expression, immune signaling pathways, and KEGG pathway analysis.

When taken as a whole, these studies demonstrate how multi-omics research is changing and how it may change how we understand complicated liver diseases by opening a new possibility for tailored medicine and more focused therapeutic interventions.

## Statistical and machine learning approaches in multi-omics HCC studies

In particular, we focused on identifying the computational strategies employed in these studies, including supervised and unsupervised machine learning approaches, to evaluate their role in biomarker discovery, patient stratification, and data integration. A pivotal part of multi-omics studies is the analysis approach applied to derive biological and clinical insights from complex and high dimensional datasets. We conducted the methods based systematic review of the 32 eligible HCC studies, in order to evaluate the computational and statistical approaches used. It was not our aim to focus on well-established procedures (e.g., the comparison would not have been valid), but rather to look for the common thread, critically appraise the rigor of the methodology and report on where the current procedure might be failing, in particular in integration, interpretability, and validation.

**Fig. 6:**
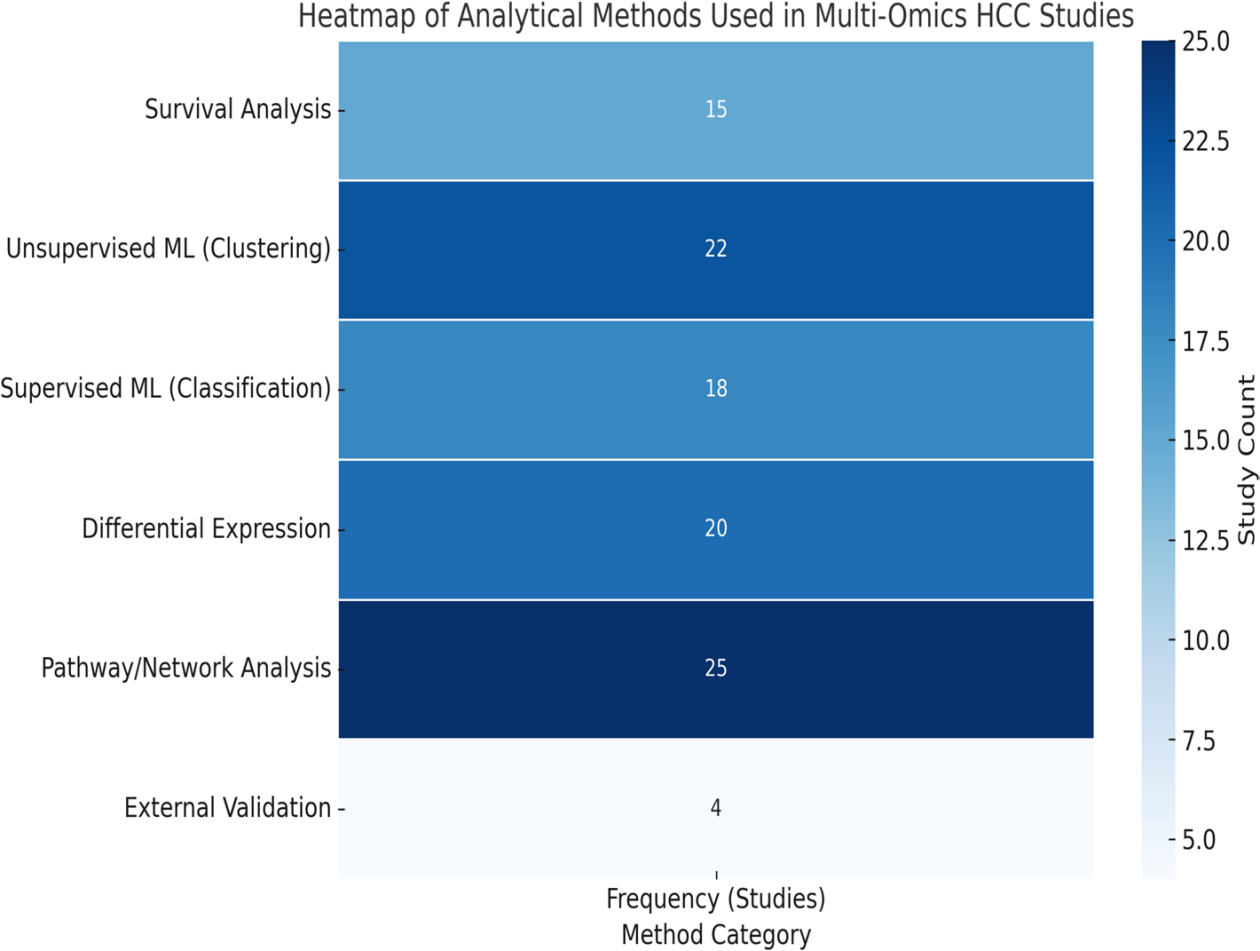
A heatmap of how often each method type was reported in the included studies

We accomplished this by grouping the reported approaches into five general categories: survival analysis, unsupervised clustering, supervised machine learning, differential expression analysis, and pathway/network analysis. We also investigated the external validation which is so important for the guarantee of model generalization. Table 5, and Fig. 6 shows a heatmap of how often each method type was reported in the included studies. Our synthesis demonstrates the primacy of unsupervised learning and pathway analysis, as well as the underuse of external validation and emergent methods like graph neural networks or causal inference.

**Table 5:** Frequency and Percentage of Analytical Methods Used in Multi-Omics HCC Studies

Method category	Frequency (studies)	Percentage (%)
Pathway/network analysis	25	78.1%
Unsupervised ML (clustering)	22	68.8%
Differential expression	20	62.5%
Supervised ML (classification)	18	56.3%
Survival analysis	15	46.9%
External validation	4	12.5%

Among the studies analyzed, machine learning methods were widely used—unsupervised clustering appeared in 68.8% of studies and supervised learning in 56.3%—highlighting their importance in subtype classification, risk prediction, and integrative analysis.

## Challenges and future directions

Despite substantial advancements, multi-omics research faces persistent challenges that hinder its effective clinical translation. A core obstacle lies in the integration of heterogeneous omics layers—such as genomics, transcriptomics, proteomics, and metabolomics which vary widely in data scale, format, and biological meaning. For instance, Chen et al. (2020) reported difficulties aligning transcriptomic and proteomic datasets due to inconsistent temporal dynamics and batch effects, leading to reduced statistical power in liver cancer subtype discovery [94].

While machine learning (ML) and network-based approaches have emerged to address these issues, most studies lack external validation and exhibit limited model interpretability. Requena et al. (2021) demonstrated this in their pan-cancer analysis where clustering based on multi-omics data was robust internally but failed to generalize across independent cohorts due to sample variability and population-specific bias [95]. Furthermore, a review by Hasin et al. (2017) emphasized that fewer than 30% of multi-omics studies in cancer integrate more than two omics layers or validate predictions with orthogonal datasets, underscoring the reproducibility gap [96].

Additionally, the lack of standardized protocols for sample collection, preprocessing, normalization, and batch correction impedes cross study comparison. Liu et al. (2019) showed how divergent sample preparation protocols in HCC proteomics studies led to non-overlapping biomarker sets, undermining reproducibility and meta-analyses [97]. Although frameworks such as FAIR data principles have been proposed, adoption is uneven—particularly in studies from resource-limited regions or using proprietary platforms.

From a modeling perspective, many integrative pipelines still favor predictive accuracy over biological interpretability, resulting in statistically sound yet mechanistically opaque findings. Zhu et al. (2022) highlighted that deep learning models applied to spatial omics data in liver tumors identified novel clusters but failed to link them to known signaling pathways, limiting their translational utility [98].

Moreover, current AI/ML models often rely on small, homogeneous datasets, rarely including early-stage disease cases or underrepresented populations. This limits the generalizability and fairness of predictive tools, as illustrated by Pan et al. (2021), who found that risk scores derived from multi-omics models performed poorly in non-Asian HCC cohorts [99]. Integrating novel technologies like single-cell sequencing and spatial omics offers higher resolution but introduces new layers of computational complexity. Pipelines such as Seurat and Scanpy, while widely used, require significant computational resources and lack interoperability with bulk omics platforms, limiting broader adoption.

From a translational standpoint, longitudinal integration of omics with clinical data remains limited. Ye et al. [100], explored spatiotemporal tumor niches in HCC and highlighted the value of longitudinal insights, yet such studies remain rare due to cost, patient follow-up constraints, and data governance issues. Ethical and regulatory concerns, especially those related to GDPR and HIPAA compliance—further complicated data sharing across institutions. Finally, while federated learning and privacy-preserving AI show promise, their application in liver cancer multi-omics remains in its infancy. Addressing these multi-level barriers will be crucial to realizing the full potential of precision oncology.

## Research gaps and future directions

Despite significant advances in applying multi-omics approaches to liver cancer research, progress is impeded by the lack of standardized protocols and benchmarking frameworks. Differences in sample processing, data acquisition, and analytical pipelines produce outputs that are difficult to compare across studies. This inconsistency undermines reproducibility and the validation of molecular signatures or biomarkers. Addressing this gap requires the development of common standards, guidelines, and reference datasets to enable robust cross-study comparisons and validation of findings. Establishing consensus protocols and community-driven benchmarks will enhance the reliability and utility of multi-omics data in hepatology.

In parallel, the complexity of multi-omics data demands scalable and interpretable computational models. High-throughput sequencing and imaging technologies produce large, high dimensional datasets that strain traditional analytical methods. Many current tools rely on opaque machine learning algorithms, which can provide limited insights into underlying biological mechanisms. Future research should emphasize the development of algorithms that scale effectively with increasing data volume while maintaining interpretability of results. Advances in interpretable machine learning and integrative analysis platforms will be crucial for translating computational findings into meaningful biological and clinical knowledge.

Another critical gap is the availability of diverse and representative datasets. Many existing multi-omics studies in liver cancer involve relatively homogeneous patient cohorts or limited experimental models, constraining the generalizability of findings. It is essential to assemble large-scale, multi-ethnic cohorts that reflect the heterogeneity of liver cancer subtypes and etiologies. Comprehensive sampling across different populations, disease stages, and risk factors will improve the robustness of multi-omics signatures and ensure that discoveries are broadly applicable. Collaborative data-sharing initiatives and open-access repositories will play an important role in assembling such diverse datasets.

Furthermore, integrating spatial and longitudinal omics data represents a promising but underexplored frontier in liver cancer research. Spatially resolved techniques, such as imaging mass cytometry and spatial transcriptomics, provide context on the tumor microenvironment, whereas longitudinal sampling of patients can capture molecular changes over time or in response to therapy. Combining these dimensions with conventional bulk and single-cell multi-omics data could reveal dynamic patterns of tumor heterogeneity, progression, and treatment response. Developing computational frameworks to integrate spatially and temporally resolved data will enable a deeper understanding of liver cancer biology and improve the modeling of disease evolution.

Finally, there is a crucial need for frameworks to translate multi-omics insights into clinical practice. Despite the wealth of molecular data generated, moving from discovery to clinical applications requires rigorous validation, clear regulatory standards, and practical implementation strategies. This translational gap necessitates interdisciplinary collaboration among computational scientists, clinicians, and regulatory experts to design studies assessing the clinical utility of multi-omics approaches. Establishing guidelines for clinical-grade data quality and interpretation, as well as conducting prospective clinical trials that incorporate multi-omics biomarkers, will be essential steps toward realizing the promise of precision hepatology.

## Conclusion

Hepatocellular carcinoma (HCC) remains a significant global health burden, marked by extensive molecular heterogeneity and poor clinical outcomes. The application of multi-omics technologies encompassing genomics, transcriptomics, proteomics, metabolomics, and more recently, single-cell and spatial omics has profoundly enhanced our understanding of liver cancer biology. These approaches have facilitated the identification of candidate biomarkers, actionable therapeutic targets, and pathways underlying drug resistance and tumor evolution.

This review highlights that while methodological advances in multi-omics and AI-driven analysis are accelerating, their clinical applicability remains constrained. Key challenges include data heterogeneity, lack of standardization in data processing, limited model interpretability, and underrepresentation of diverse patient populations. Moreover, many studies emphasize computational novelty over translational relevance, resulting in models with limited generalizability to real-world settings.

Despite these barriers, the field is moving toward greater integration of multi-omics data with clinical metadata, offering promise for more personalized and adaptive treatment strategies. Advances in federated learning, privacy preserving AI, and ethical data governance frameworks are beginning to address privacy, and interoperability concerns critical to large scale implementation.

To fully realize the potential of multi-omics in liver cancer, future research must prioritize:

Longitudinal and spatially resolved studies to capture tumor heterogeneity over time and within microenvironments; Robust validation across diverse populations to ensure equity and generalizability; Standardized pipelines and FAIR data practices to enhance reproducibility and collaboration; and most critically, interdisciplinary efforts that bridge computational innovation with clinical need. In conclusion, multi-omics is poised to transform the diagnosis, prognosis, and treatment of liver cancer. Its successful translation into the clinic will depend not only on technological sophistication, but on collaborative, ethically grounded strategies that make precision oncology a reality for all patients.

## Data Availability

No datasets were generated or analysed during the current study.
